# The D299G/T399I Toll-Like Receptor 4 Variant Associates with Body and Liver Fat: Results from the TULIP and METSIM Studies

**DOI:** 10.1371/journal.pone.0013980

**Published:** 2010-11-15

**Authors:** Peter Weyrich, Harald Staiger, Alena Stančáková, Fausto Machicao, Jürgen Machann, Fritz Schick, Norbert Stefan, Johanna Kuusisto, Markku Laakso, Silke Schäfer, Andreas Fritsche, Hans-Ulrich Häring

**Affiliations:** 1 Division of Endocrinology, Diabetology, Vascular Disease, Nephrology, Clinical Chemistry, Department of Internal Medicine, Paul-Langerhans-Institute Tübingen, University of Tübingen, Tübingen, Germany; 2 Department of Medicine, Kuopio University Hospital, Kuopio, Finland; 3 Section on Experimental Radiology, University of Tübingen, Germany; University of Bremen, Germany

## Abstract

**Background:**

Toll-like-receptor 4 (TLR) is discussed to provide a molecular link between obesity, inflammation and insulin resistance. Genetic studies with replications in non-diabetic individuals in regard to their fat distribution or insulin resistance according to their carrier status of a common toll-like receptor 4 (TLR4) variant (TLR4^D299G/T399I^) are still lacking.

**Methodology/Principal Findings:**

We performed a cross-sectional analysis in individuals phenotyped for prediabetic traits as body fat composition (including magnetic resonance imaging), blood glucose levels and insulin resistance (oral glucose tolerance testing, euglycemic hyperinsulinemic clamp), according to TLR4 genotype determined by candidate SNP analyses (rs4986790). We analyzed N = 1482 non-diabetic individuals from the TÜF/TULIP cohort (South Germany, aged 39±13 y, BMI 28.5±7.9, mean±SD) and N = 5327 non-diabetic participants of the METSIM study (Finland, males aged 58±6 y, BMI 26.8±3.8) for replication purposes. German TLR4^D299G/T399I^ carriers had a significantly increased body fat (*XG* in rs4986790: +6.98%, *p* = 0.03, dominant model, adjusted for age, gender) and decreased insulin sensitivity (*XG*: −15.3%, Matsuda model, *p* = 0.04; *XG*: −20.6%, *p* = 0.016, clamp; both dominant models adjusted for age, gender, body fat). In addition, both liver fat (*AG*: +49.7%; *p* = 0.002) and visceral adipose tissue (*AG*: +8.2%; *p* = 0.047, both adjusted for age, gender, body fat) were significantly increased in rs4986790 minor allele carriers, and the effect on liver fat remained significant also after additional adjustment for visceral fat (*p* = 0.014). The analysis in METSIM confirmed increased body fat content in association with the rare *G* allele in rs4986790 (*AG*: +1.26%, *GG*: +11.0%; *p* = 0.010, additive model, adjusted for age) and showed a non-significant trend towards decreased insulin sensitivity (*AG*: −0.99%, *GG*: −10.62%).

**Conclusions/Significance:**

TLR4^D299G/T399I^ associates with increased total body fat, visceral fat, liver fat and decreased insulin sensitivity in non-diabetic Caucasians and may contribute to diabetes risk. This finding supports the role of TLR4 as a molecular link between obesity and insulin resistance.

## Introduction

Obesity and excess of saturated free fatty acids (SFFAs) cause low-grade inflammatory processes with subsequent development of insulin resistance, which is a major risk factor for later onset of type 2 diabetes mellitus [1]. As the prevalence of obesity and type 2 diabetes mellitus (T2DM) increases throughout the world with remarkable socio-economic impact [2], it is important to understand the molecular mechanisms that link dietary SFFA intake and endogenous SFFAs derived from adipose tissue lipolysis, respectively, to inflammation.

In this context, toll-like receptor (TLR) signaling moved into the center of current scientific debate [3]. TLRs are transmembrane receptors which play a crucial role in the innate, non-adaptive immune system. The human TLR family comprises at least 13 distinctive proteins (TLR1 – TLR13) that are able to recognize typical microbial agents and subsequently facilitate an early immune response via downstream signaling to different signaling pathways (see [4], [5], [6] for review). The human TLR member characterized best to date is toll-like receptor 4 (TLR4; NM_138554; OMIM 603030), as this signaling molecule is essential for the recognition of bacterial lipopolysaccharides (LPS) beyond other microbial agents [7]. LPS represent a key element of gram-negative bacteria and contain fatty acids in their lipid-A domain anchoring LPS into the bacterial cell wall [8]. First evidence that TLR4 not only acts for LPS as a signaling receptor but also for other lipids as e.g. SFFAs came up from studies in macrophages expressing TLR4 mutants [9]. Subsequent studies revealed that TLR4 signaling actually is essential for the development of SFFA-induced insulin resistant states in different cellular models, as e.g. in 3T3L1-adipocytes [10], [11] and L6 myotubes [12]. It was also shown that TLR4 mediates the crosstalk between macrophage-induced adipose tissue lipolysis and increased inflammatory activity in both adipocytes and macrophages [13]. In addition, TLR4^−/−^ mice are protected against down-regulation of insulin signaling in skeletal muscle upon systemic lipid infusion [11], [12], and C3H/HeJ mice bearing a loss-of-function mutation in TLR4 do not develop diet-induced obesity or insulin-resistance in skeletal muscle [14], nor do they develop dietary hepatic steatosis [15]. A very recent molecular study provides further insights into the molecular events occurring after SFFA-induced TLR4 receptor stimulation, as both TLR4 dimerization and its recruitment to lipid-rafts seem to be influenced by SFFAs [16]. In humans, obese and type 2 diabetic subjects show both an increased *TLR4* gene expression and TLR4-dependent activation of the inflammatory signaling molecules IκB/NFκB [17], and dietary restriction results in down-regulation of *TLR4* expression [18].

In summary, there is evidence that TLR4 may provide the molecular mechanism that links increased SFFAs in obese subjects to the observed chronic low-grade inflammatory state and subsequent manifestation of insulin resistance. Further evidence for the potential in-vivo relevance of this mechanism would result from genetic studies in populations with common TLR4 mutants. In this regard, two SNPs (rs4986790, rs4986791) in exon 3 of the TLR4 gene (*TLR4*) causing single amino acid replacements (rs4986790: D299G; rs4986791: T399I) in the extracellular TLR4 domain were shown to be present at allele frequencies between 0.03 and 0.05 in different Caucasian populations [19]. In Europeans, co-segregation (D' = 1.0, r^2^ = 1.0) of both SNPs occurs [20], probably owing to genetic drift after selection of the TLR4^D299G^ mutant in Africa which confers resistance to malaria [21]. As the two non-synonymous SNPs rs4986790/91 in the extracellular domain of TLR4 may bear functional consequences on ligand-binding, many studies focused on the relevance of rs4986790/91 on both the cellular level and in genetic association studies, revealing conflicting results [22].

Most geneticists focused on immunologic and inflammatory phenotypes associating with the TLR4^D299G/T399I^ variant [23], [24], [25], [26], [27], [28], and only a limited number of studies addressing the role of TLR4^D299G/T399I^ for diabetic traits are available. While an early study was not able to verify the association of TLR4^D299G/T399I^ with changes in diabetes prevalence [29], a recent work supports that male Caucasian TLR4^D299G/T399I^ individuals are at higher T2DM risk, dependent on the ratio between (total):(high-density-lipoprotein) cholesterol levels [30]. In addition, increased risk of early onset of diabetic retinopathy was reported for TLR4^D299G/T399I^ carriers in Brasil [31]. On the other hand, the prevalence of diabetic neuropathy seemed to be lower in TLR4^D299G/T399I^ Caucasian individuals [32], and protection from both T2DM [33] and the metabolic syndrome [34] by TLR4^D299G/T399I^ also has been described.

To our knowledge, there are no investigations that have addressed potential prediabetic phenotypes in carriers of the TLR4^D299G/T399I^ variant. We, therefore, conducted this genetic association study on TLR4^D299G/T399I^ in non-diabetic subgroups of two independent Caucasian populations, namely the TÜF/TULIP cohort (South Germany) and the METSIM cohort (Finland). We focused on both body composition parameters as well as insulin resistance in light of above listed TLR4 signaling functions.

## Materials and Methods

### Study Participants

TÜF/TULIP cohort – 1635 participants of the Tübingen Family Study (TÜF) and the Tübingen Lifestyle Intervention Program (TULIP) in South West Germany were analyzed for this study. All volunteers signed informed written consent to the study protocol which was approved by the local medical ethics committee. 153 subjects had to be excluded owing to manifest diabetes or missing data resulting in a total cohort of n = 1482 non-diabetic individuals. The participants (see Table 1 for additional characteristics) did not take any medication known to affect glucose tolerance, and strict abstention from smoking was demanded 24 h before and during the test period including an oral glucose tolerance test (OGTT; all participants, see below) and a hyperinsulinaemic-euglycaemic clamp (N = 502 participants). As described elsewhere [35], the aim of the TULIP study is to provide a broad range of metabolic data of non-diabetic individuals with increased diabetes risk, as e.g. a positive family history of diabetes, a BMI of >27 kg/m^2^, the presence of impaired fasting glucose (IFG) or former gestational diabetes.

**Table 1:** Clinical characteristics of the TÜF-TULIP and METSIM study population.

	TÜF-TULIP	METSIM
Gender (female/male)	978/504	0/5327
IFG/IGT/(IFG+IGT)	48/193/59	884/503/346
Age (y)	39±13	58±6
BMI (kg/m^2^)	28.5±7.9	26.8±3.8
Body Fat (%)	30.4±10.4	23.9±6.5
Fasting glucose (mmol/l)	5.11±0.57	5.68±0.50
Glucose, 120 min OGTT (mmol/l)	6.26±1.67	6.09±1.69
Fasting insulin (pmol/l)	62.2±51.1	48.6±33.7
Insulin, 30 min OGTT (pmol/l)	424±443	391±284

Data are given as means ±SD. BMI – body mass index; IFG – impaired fasting glucose (defined as 100–126 mg/dl according to AHA guidelines); IGT – impaired glucose tolerance; OGTT – oral glucose tolerance test.

METSIM cohort – The METabolic Syndrome In Men (METSIM) Study is a population-based random sample of 6229 Finnish men aged from 45 to 73 years in Eastern Finland (Kuopio, see Table 1) who were phenotyped during a one-day visit to the Clinical Research Unit of the University of Kuopio for diabetes risk and cardiovascular disease variables. All study participants underwent an OGTT, and the study protocol was approved by the local ethics committee. For this study, only non-diabetic METSIM participants were analyzed (N = 5327). However, additional inclusion of METSIM participants with diagnosed diabetes (N = 898) according to the WHO criteria [36] allowed estimation for diabetes risk using logistic regression analysis in a separate analysis.

### Analytical procedures

A bedside glucose analyzer (Yellow Springs Instruments, Yellow Springs, OH, USA) was used to measure blood glucose. Plasma insulin was determined by commercial chemiluminescence assays from ADVIA Centaur (Siemens Medical Solutions, Fernwald, Germany; unit: pmol/l). In the METSIM Study, plasma glucose was measured by enzymatic hexokinase photometric assay (Konelab Systems Reagents, Thermo Fischer Scientific, Vantaa, Finland), and insulin was determined by immunoassay (ADVIA Centaur Insulin IRI, no 02230141, Siemens Medical Solutions Diagnostics, Tarrytown, NY; unit: mU/l) according to the manufacturer's instructions.

### Oral glucose tolerance test (OGTT)

TÜF/TULIP and METSIM participants were investigated in the fasting state at baseline. Subsequently, all participants underwent an OGTT performed according to the WHO recommendations [37]. Blood glucose and plasma insulin were determined at 0, 30, 60, 90 and 120 min in the TÜF/TULIP Study and at 0, 30, and 120 min in the METSIM Study.

### Determination of body and liver fat

The body mass index (BMI) was calculated as weight divided by the square of height (kg/m^2^). Body fat was determined by bioelectrical impedance (RJL; Detroit, MI, USA). In the TULIP cohort, liver fat was determined by magnetic resonance spectroscopy (1.5 T Magnetom Sonata; Siemens, Erlangen, Germany) in the posterior 7^th^ segment of the liver. A single-voxel stimulated echo acquisition mode technique was used (repetition time = 4 s, echo time = 10 msec, 32 acquisitions) for a voxel of 3×3×2 cm^3^, and liver fat content was calculated by the signal integral (methylene/methyl signals between 0.7–1.5 ppm) in reference to the sum of water and lipid signal integrals [38], [39].

### Insulin sensitivity

Insulin sensitivity was estimated using the Matsuda model [40] in both study cohorts, based on plasma glucose and plasma insulin levels obtained at 0, 30, 60, 90 and 120 min in TÜF/TULIP and 0, 30, 120 min in METSIM, respectively. In the METSIM cohort, we additionally used the Homeostasis Model Assessment of Insulin Resistance (HOMA-IR) for estimation of insulin resistance [41]. In N = 502 TÜF/TULIP participants, an additional hyperinsulinaemic euglycaemic clamp was undertaken during a second visit. Further details on the clamping technique are described elsewhere [42].

### Genotyping

The SNP rs4986790 (exon 3) encoding the D299G mutation in the extracellular domain of TLR4 was genotyped by use of the TaqMan assay (Eurogentec; Liege, Belgium) and the fluorescence detecting ABI Prism 7500 (Applied Biosystems; Foster City, CA, USA). To verify the reported co-segregation of rs4986790 (exon 3, D299G) with rs4986791 (exon3, T399I; D' = 1.0, r^2^ = 1.0 in the CEU population, HapMap Rel #24 PhaseII Nov08 [20]) and TaqMan validity in our cohort, we sequenced for both SNPs in N = 388 German participants, verifying the reported linkage disequilibrium (D' = 1.0, r^2^ = 1.0). Genotyping quality was ensured by including three sequenced controls of each rs4986790 genotype in the TaqMan assay plates.

### Statistics

The JMP 4.0.4 software (SAS Institute; Cary, NC, USA) and SPSS 14.0 for Windows (SPSS Inc., Chicago, IL, USA) were used for statistical analysis. Data are presented as means ± SD if not otherwise stated. The χ^2^-test was used for testing Hardy-Weinberg-Equilibrium. All non-normally distributed parameters were logarithmically transformed before analysis by ANOVA or *t*-tests without adjustment or with adjustment to relevant covariates in linear regression models. A *p* value≤0.05 was considered statistically significant.

## Results

### Distribution of D299I-TLR4 genotypes

The genotype call rate for rs4986790 was 98.2/100% in the TÜF/TULIP and the METSIM Study, respectively. The minor allele frequency of rs4986790 was 0.052 in TÜF/TULIP and 0.094 in METSIM, and both genotype distributions were in Hardy-Weinberg-Equilibrium (TÜF/TULIP: *p* = 0.86; METSIM: *p* = 0.64).

### D299G-TLR4 association analyses in TÜF/TULIP

Associations in this population were only calculated based on a dominant model owing to the low minor allele frequency of rs4986790 in TÜF/TULIP, resulting in 3 homozygote minor allele carriers. The minor allele carriers of rs4986790 had a significantly increased body fat (e.g. *XG*: +6.98%, *p* = 0.03 in the dominant model adjusted for age and gender, see Table 2), and an allele-dose effect on body fat could be assumed (*AG*: +6.9%, *GG*: +9.2%; see also results of METSIM below). There was no significant association between rs4986790 genotype and BMI or blood glucose (both 0 and 120 min OGTT) levels (*p*>0.06; all). In contrast, insulin sensitivity was significantly decreased in rs4986790 minor allele carriers, independently of whether the Matsuda estimation model (*XG*: −15.3%) or insulin sensitivity data measured by clamp (*XG*: −20.6%) were used for analysis. This association remained significant after adjustment for age, gender and BMI (see Table 2) or body fat (Matsuda: *p* = 0.04; clamp: *p* = 0.016; dominant model). We additionally stratified the TÜF/TULIP cohort for gender, as the METSIM cohort only includes male participants. While the association with body fat (*p* = 0.04, adjusted for age; dominant model) and insulin sensitivity (Matsuda: *p* = 0.002; clamp: *p* = 0.013, both adjusted for age, gender and BMI; dominant model) was more significant in males, both association analyses turned out to be non-significant in women (body fat: *p* = 0.24, Matsuda/clamp: *p*>0.53).

**Table 2:** Associations of *TLR4* SNP rs4986790 (D299G) with anthropometric and metabolic data in the TÜF-TULIP cohort (N = 1482).

SNP	rs4986790			dominant model	
Genotype	AA	AG	GG	p_1_	p_2_
N	1330	149	3		
Age (y)	39±13	39±13	29±3	0.86	0.96
BMI (kg/m^2^)	28.4±7.8	29.5±8.6	29.9±10.2	0.11	0.13
Body Fat (%)	30.2±10.4	32.3±10.7	33.0±18.0	**0.02**	**0.03**
Fasting glucose (mM)	5.11±0.57	5.17±0.58	4.84±0.15	0.25	0.42
Glucose, 120 min OGTT (mM)	6.23±1.67	6.48±1.59	7.30±1.92	0.06	0.09
Insulin sensitivity (Matsuda), OGTT (AU)	16.8±11.0	14.26±9.0	11.33±6.1	**0.004**	**0.03**
Insulin sensitivity, clamp (U)*	0.087±0.056	0.069±0.046	0.069±0.069	**0.026**	**0.04**

Data are given as means ±SD. **p_1_** – non-adjusted model; **p_2_** – adjusted model with log-transformed data; age was adjusted for gender; BMI and body fat were adjusted for gender and age; glucose levels as well as insulin sensitivity (Matsuda estimation model, clamp) were adjusted for gender, age, and BMI; BMI – body mass index; OGTT – oral glucose tolerance test; SNP – single nucleotide polymorphism.

*N = 502.

**Figure 1:**
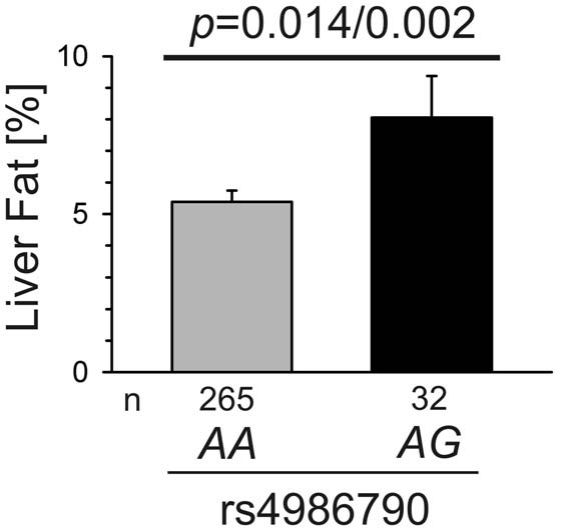
Liver fat according to D299G-TLR4 (rs4986790) genotype. Liver fat was determined by magnetic resonance imaging in the German cohort in 297 participants. Indicated *p*-values refer to the non-adjusted/adjusted model, with adjustments for age, gender and BMI (alternative adjustment for body fat did not alter significance). Data are presented as mean +SEM.

As liver fat is an important determinant for insulin resistance [43], we subsequently analyzed whether the rs4986790 genotype also associates with liver fat measured by magnetic resonance imaging (MRI). Indeed, liver fat was significantly increased in heterozygous rs4986790 minor allele carriers (*AG*: +49.7%; N = 297, no liver fat data from homozygous carriers available; see Figure 1). In addition, visceral adipose tissue (VAT) content in rs4986790 SNP carriers was significantly higher (*AG*: +8.2%; p = 0.047, adjusted for age, gender and body fat) in the subgroup where VAT was determined by MRI (N = 315, no homozygous genotypes available, data not shown). The liver fat analysis remained significant after adjustment for VAT (*p* = 0.014, adjusted for age, gender, body fat and VAT).

### D299I-TLR4 association analyses in METSIM

The association of rs4986790 genotype with body fat was reproducible in METSIM (*AG*: +1.26%, *GG*: +11.0%; *p* = 0.010, additive model, see Table 3). Similarly as in TÜF/TULIP, BMI and blood glucose levels were not associated with the rs4986790 genotype (*p*>0.10; all). Insulin sensitivity (Matsuda model) also decreased in rs4986790 *G* allele carriers (*AG*: −0.99%, *GG*: −10.50%, see Table 3), and insulin resistance (HOMA-IR) accordingly increased (*AG*: +0.96%, *GG*: +2.07%, see Table 3). However, this effect remained non-significant, independent of the used genetic calculation model or adjustment to BMI (or body fat) and age (*p*>0.21, all). Logistic regression analysis comparing diabetic with non-diabetic METSIM study participants did not reveal a significantly altered diabetes prevalence in rs4986790 minor allele (p>0.264, additive and dominant model). In contrast to the findings of Kolz et al. [30], interaction analysis of TLR4 rs4986790 genotype by the ratio of total-cholesterol:HDL-cholesterol (TC/HDL-C) was not significant (*p* = 0.28; dominant model). Stratification of the METSIM cohort for TC/HDL-C was, therefore, not admissible.

**Table 3:** Associations of *TLR4* SNP rs4986790 (D299G) with anthropometric and metabolic data in the METSIM cohort (N = 5327).

SNP	rs4986790			additive		dominant	
Genotype	AA	AG	GG	p_1_	p_2_	p_1_	p_2_
N	4375	902	50				
Age (y)	58±6	59±7	59±7	0.57	-	0.29	-
BMI (kg/m^2^)	26.9±3.8	26.7±3.8	27.8±3.7	0.10	0.10	0.58	0.59
Body Fat (%)	23.9±6.4	24.2±6.6	26.5±6.7	**0.015**	**0.010**	0.10	0.19
Fasting glucose (mM)	5.68±0.50	5.69±0.49	5.71±0.53	0.77	0.67	0.48	0.41
Glucose, 120 min OGTT (mM)	6.08±1.68	6.13±1.74	6.33±1.65	0.43	0.61	0.39	0.38
Insulin sensitivity (Matsuda), OGTT (AU)	7.05±4.19	6.97±4.14	6.31±3.27	0.61	0.36	0.46	0.21
Insulin resistance index (HOMA-IR), OGTT (AU)	2.078±1.509	2.080±1.535	2.121±1.335	0.83	0.52	0.76	0.38

Data are given as means ±SD. **p** values were calculating using log-transformed variables. **p_1_** – non-adjusted model; **p_2_** – adjusted model as follows: BMI and body fat were adjusted for age; glucose levels as well as insulin sensitivity (Matsuda estimation model; Homeostasis Model Assessment of Insulin Resistance: HOMA-IR) were adjusted for age and BMI; BMI – body mass index; OGTT – oral glucose tolerance test; SNP – single nucleotide polymorphism.

## Discussion

This is the first study to have investigated the role of the TLR4^D299G/T399I^ double mutant for body fat composition and insulin resistance in non-diabetic populations at increased diabetes risk. Our finding is a consistent association of the minor allele of the selected tagging SNP rs4986790 with increased body fat in both the TÜF/TULIP and the METSIM study cohort. The effect on body fat was remarkable in both populations, especially in homozygous minor allele rs4986790 carriers (+9.2% and +11.0%, respectively). In the German cohort, there were also significant associations of the minor *G* allele in rs4986790 with increased insulin resistance, increased liver fat and increased visceral fat in adjusted linear models. In METSIM, there was only a non-significant trend to reduced insulin sensitivity. We assume that this finding may be a consequence of the different structure of both study populations. In METSIM, participants were selected randomly from the population of Kuopio and are older by an average of 20 years, while TÜF/TULIP comprises mainly non-diabetic individuals at a younger age, selected based on the assumption of an increased diabetes risk.

The link between the innate immune system and obesity comes from common features of both macrophages and adipocytes. Both cell types secrete various pro-inflammatory chemokines and cytokines and intensely interact yet due to their close physical location in adipose tissue [44], [45], and both adipocytes and macrophages express TLR4 on their plasma membrane [4], [10]. TLR4 expression in visceral adipose tissue (VAT) of obese subjects exceeds that of the subcutaneous fat compartment, in contrast to lean individuals, where TLR4 is expressed more in subcutaneous fat compared to VAT [46]. A recent study also showed that TLR4 expression is increased on VAT-derived macrophages from both obese and diabetic individuals, and that both adipocyte size and systemic CRP levels are increased according to macrophage infiltration and TLR4 expression [47]. Taken together, these data yet support our finding that a TLR4 variant impacts on body fat composition and insulin resistance.

Above mentioned studies let assume that the TLR4^D299G/T399I^ double mutant confers an intrinsic activation to TLR4, at least in adipose tissue and liver fat. However, functional assays with TLR4^D299G/T399I^ revealed inconclusive results. While some authors reported hypo-responsiveness of this TLR4 mutant to stimuli like e.g. LPS [25], other groups could not verify any differences in receptor functionality comparing TLR4^D299G/T399I^ to wildtype TLR4 [27]. Therefore, future studies should address adipocyte TLR4 signaling in adipose tissue samples derived from TLR4^D299G/T399I^ carriers to finally address this issue.

One could speculate that differences in serum free fatty acid (FFA) levels may have contributed to the reported phenotype with higher body fat content. However, serum FFA levels did not differ in TÜF/TULIP according to TLR4 genotype, and systemic cytokines (TNFα, IL6) as a possible readout of inflammatory activity were also not altered significantly (data not shown). As the effect on liver fat was independent of the increased VAT content in German TLR4^D299G/T399I^ carriers, we assume that the observed increase in ectopic hepatic fat may be a consequence from altered direct crosstalk between hepatic macrophages and adipocytes, and not an epiphenomen caused by potential VAT mediator release. It is well known that liver fat generally associates with insulin resistance [48], [49], but we also have learned that this association is subject to remarkably high variability [43]. Taking into account that the increase in liver fat in TLR4^D299G/T399I^ carriers comes along with decreased insulin sensitivity in TÜF/TULIP, one may speculate whether TLR4 may represent a candidate for the mediation of metabolically unfavorable effects of liver fat.

The limitation of our study is that Bonferroni's correction for 3 independent endpoints (body fat, plasma glucose, insulin sensitivity, expected α-level: 0.017) would render the reported finding for body fat composition nominal. In addition, the increase in body fat in non-diabetic individuals was not reflected by a significant increase in diabetes prevalence in the METSIM study.

In summary, we conclude that the TLR4^D299G/T399I^ double mutant is associated with increased body fat and insulin resistance. Due to its low allele frequency throughout different populations [21], TLR4^D299G/T399I^ cannot be seen as a strong obesity or diabetes risk gene, but may contribute to factors that are well known to entail a relevant predisposition to later onset of type 2 diabetes mellitus.
